# Supplementary material for: A Critical View of the Application of the APEX Software (Aqueous Photochemistry of Environmentally-Occurring Xenobiotics) to Predict Photoreaction Kinetics in Surface Freshwaters
Source: Molecules. 2019 Dec 18;25(1):9. doi: 10.3390/molecules25010009 (PMC7017383; doi:10.3390/molecules25010009)
Supplement: Supplementary file 1 [file molecules-25-00009-s001.zip › 3.2.4_gcc-4.4.0.zip!/3.2.4_gcc-4.4.0/3.2.4_gcc-4.4.0/doc/octave/html/interpreter/Format-of-Descriptions.html]

Format of Descriptions - Untitled


Previous: Error Messages,
Up: Conventions


---

#### 1.3.5 Format of Descriptions

Functions, commands, and variables are described in this manual in a
uniform format. The first line of a description contains the name of
the item followed by its arguments, if any.
The category—function, variable, or whatever—appears at the
beginning of the line.
The description follows on succeeding lines, sometimes with examples.

- A Sample Function Description- A Sample Command Description- A Sample Variable Description
